# Self-referrals in the emergency department: reasons why patients attend the emergency department without consulting a general practitioner first—a questionnaire study

**DOI:** 10.1186/s12245-015-0096-x

**Published:** 2015-12-07

**Authors:** Nicole Kraaijvanger, Douwe Rijpsma, Henk van Leeuwen, Michael Edwards

**Affiliations:** Emergency Department, Rijnstate Hospital, Wagnerlaan 55, Arnhem, The Netherlands; Department of Internal Medicine/Intensive Care, Rijnstate Hospital, Wagnerlaan 55, Arnhem, The Netherlands; Trauma Surgery/Emergency Department, Radboud University Medical Centre, Geert Grooteplein Zuid 10, Nijmegen, The Netherlands

**Keywords:** Emergency department, Self-referrals, Motives, Appropriateness, Questionnaires

## Abstract

**Background:**

To influence self-referral, it is crucial to know a patient’s motives to directly visit the emergency department (ED). The goal of this study is to examine motives for self-referral to the ED and compare these motives in relation to appropriateness.

**Methods:**

All self-referred patients visiting the ED of a Dutch hospital over four separate months in a 1-year period were included. Patients were handed questionnaires that included questions on their reasons to visit the ED directly and where they would seek medical help next time. Additionally, the motives of patients that either appropriately or inappropriately visited the ED were compared. In a previous study on the same patient cohort, the appropriateness of the ED visits was determined using predefined criteria.

**Results:**

A total of 3196 self-referred patients were included, and 48.9 % completed the questionnaires. The majority of patients (28.0 %) attended the ED without a referral because they thought they would get help faster; the next reason was the easier access to radiologic and laboratory investigations (answered by 23.8 %); and the third was the symptoms were considered too severe to visit a general practitioner (GP) (answered by 22.7 %). The majority (78.5 %) would attend the ED the next time they are faced with similar symptoms. Appropriate visits were significantly more seen in females, elderly, and patients in higher triage categories. Patients who expect investigations are necessary, think their symptoms are too severe to visit a GP, or would return to the ED next time were more often appropriately visiting the ED.

**Conclusions:**

The choice of patients to self-refer to an ED is often an explicate decision. Patients are looking for specialist help and want fast and easy access to radiologic and laboratory investigations. Even though the primary care network is well developed in the Netherlands, the reasons for self-referral are similar to the reasons found in previous literature based in other countries. Patients who visit the ED because of health concerns visit the ED more often appropriately than patients visiting for practical reasons.

## Background

The question of inappropriate self-referrals to the emergency department (ED) is contentious and continues to provoke discussion in the light of increasing healthcare expenses and crowding.

The healthcare system in the Netherlands has a strong primary care network. The general practitioner (GP) serves as a gatekeeper, referring patients with acute illnesses to the ED, only if deemed necessary. During working hours, patients can consult their own GP; in out-of-office hours, they can consult a GP at a GP cooperative. However, patients can also choose to go directly to the ED. A recent Dutch study found that self-referred visits account for an average of 30 % of all ED visits [1]. Many of these patients present with problems that possibly could be taken care of by a GP at lower costs [2–4]. Consequently, to reduce costs, policymakers in healthcare and insurance companies are investigating methods to reduce the number of self-referrals to the ED.

In the Netherlands, people are obligated to have health insurance. In 2008, a deductible of 150 € was introduced. This deductible gradually increased over the years to 375 € in 2015. When someone reaches their deductible amount, additional medical visits (including ED visits) are fully covered by medical insurance. This deductible does not apply to care provided by a GP. Despite these measures, the number of ED visits has not decreased over the past years and it is not clear whether the number of inappropriate visits has.

To influence self-referral, it is essential to know the patient’s motives to directly visit the ED, bypassing their GP. Previous studies found multiple reasons for patients to self-refer to the ED, ranging from practical issues to concerns of having a serious condition [3, 5–13]. However, most of these previous studies did not include the entire ED population or were not conducted in the Netherlands. The goal of this study is to explore the motives of self-referred patients to directly visit the ED in the Netherlands and to compare the motives of patients either appropriately or inappropriately visiting the ED.

## Methods

### Study design

This is an observational and prospective study. Self-referred patients in the ED were handed questionnaires with questions on their reasons to visit the ED. Next, predefined criteria (Table 1) were used to compare the motives of patients that either appropriately or inappropriately visited the ED.

**Table 1:** Predefined criteria determining the appropriateness of the ED visit

Secondary care (appropriate)	Primary care (inappropriate)
Laboratory investigations	Urine testing only
ECG	
Immediate radiologic investigations (X-ray, CT, ultrasound, MRI)	
Extensive wounds that needed follow-up in a specialist office	Simple suture wounds that did not need follow-up or could be followed up by a GP
Complications/symptoms related to previous hospital treatment	
Indication for surgery	
Hospital admission	

### Study setting

This study is performed in the ED of a 955-bed community teaching hospital in the Netherlands (Rijnstate Hospital) that covers an area with 460,000 inhabitants. The ED is 24/7 staffed by a team consisting of nine emergency physicians and 27 emergency medicine residents. In 2012, there were 36,721 ED visits, of which 12,383 patients (33.7 %) were hospitalized. The nearest GP cooperative is 5.6 km (3.48 miles) away.

In 2012, 93 EDs were operational 24/7 in the Netherlands. Twenty-eight EDs were in hospitals of the association of tertiary medical teaching hospitals (STZ-hospitals), as is the Rijnstate Hospital. In this category, there was an average of 31,346 visits per ED in 2012 (ranging from 17,000 to 50,000 ED visits). The average percentage of hospitalized patients in STZ-hospitals was 32 % (ranging from 8 to 43 %) [1].

### Participants

After approval from the Local Ethics Committee of the Rijnstate Hospital, all self-referred patients visiting the ED were included. Patients attending the ED on their own initiative, without a referral from a GP and not brought in by ambulance were considered ‘self-referred’. No exclusion criteria were used. To avoid bias based on seasonal variation, patients were included in four separate months (April, July and October of 2012, and January of 2013). This patient cohort was subject of a previous study, concerning the percentage of appropriate visits of self-referred patients in the ED [4].

### Questionnaires

Questionnaires were handed to the participants. When the patient was under the age of 12, caregivers were asked to fill out the questionnaire. First, they could fill in what the reason (symptom) was to attend the ED. These reasons were then coded using the ‘Reason for Visit Classification for Ambulatory Care’, developed by the US Department of Health, Education, and Welfare in 1979 [14]. Second, patients could fill in why they visited the ED directly. This was a multiple choice question, but there was a possibility to give an open answer. When patients wrote a statement that was similar to one of the multiple choice answers, it was classified as such. When patients chose more than one answer to this question, all answers were included. Third, patients could fill in where they would go the next time, confronted with similar symptoms; this was a multiple choice question. Informed consent was obtained from the participants.

### Appropriate versus inappropriate visits

The motive of self-referrals to directly visit the ED was the primary outcome of this study. In addition, the motives of patients that either appropriately or inappropriately visited the ED were compared. In a previous study, performed on the same patient cohort, the percentage of appropriate visits to the ED was determined using predefined criteria [4]. These criteria classified an ED visit as appropriate, when it warranted diagnostic testing or treatment that could only be performed in a hospital (Table 1). These criteria were applied after the primary assessment in the ED and were not known to the treating physician. The predefined criteria classified 1878 ED visits (58.8 %) as appropriate.

### Statistics

All data were analyzed in SPSS Statistics (SPSS Inc. PASW Statistics for Windows, version 19.0). Descriptive statistics were used to describe the patient population. We explored whether the appropriateness of an ED visit was related to gender, age, Manchester triage category, reasons to visit the ED directly and where patients would seek medical help next time. Differences in distributions of these categorical variables were compared using the Pearson chi-square test. A *P* value less than 0.05 was considered statistically significant. To control the false discovery rate in multiple testing (i.e. reduce the probability of type I errors), the Benjamini-Hochberg method was used.

## Results

During the inclusion period, a total of 12,409 patients attended the ED. Twenty-six percent (3196 patients) visited the ED without a referral from a GP (of which 9.4 % were hospitalized). A total of 1563 patients completed the questionnaire, which results in a response rate of 48.9 %. Of these patients, 6.2 % was hospitalized. Table 2 shows the patient characteristics and the percentages of different patient categories either appropriately or inappropriately visiting the ED. Appropriate visits were significantly more seen in female patients, elderly and higher triage categories.

**Table 2:** Patient characteristics versus appropriateness

Category	Variable	Total *N* (%)	Quest. *N* (%)	Appropr. *N* (%)	Inapp. *N* (%)	*P* value
Gender	Male	1875 (59)	932 (50)	519 (56)	413 (44)	0.019
	Female	1321 (41)	631 (48)	389 (62)	242 (38)	
Age	<18	697 (22)	337 (48)	177 (53)	160 (48)	<0.001
	18–39	1308 (41)	646 (49)	347 (54)	299 (46)	
	40–59	778 (24)	386 (50)	253 (66)	133 (35)	
	60–79	346 (11)	162 (47)	106 (65)	56 (35)	
	>80	67 (2)	32 (48)	25 (78)	7 (22)	
Manchester Triage category	Red	1 (0.03)	0 (0)	0 (0)	0 (0)	<0.001
	Orange	178 (6)	57 (32)	51 (89)	6 (11)	
	Yellow	1189 (37)	550 (46)	378 (69)	172 (31)	
	Green	1788 (56)	944 (53)	477 (51)	467 (49)	
	Blue	30 (1)	12 (40)	2 (17)	10 (83)	
	No triage	10 (0.3)	0 (0)	0 (0)	0 (0)	

*Total N (%)* number of self-referred patients per group (percentage of category), *Quest. N (%)* number of questionnaires filled out per group (percentage), *Appropr. N (%)* number of appropriate visits by patients that filled out the questionnaires per group (percentage of appropriate visits per group), *Inapp. N (%)* number of inappropriate visits by patients that filled out the questionnaires per group (percentage of inappropriate visits per group), *P value* appropriate versus inappropriate, per category

Fifteen hundred thirty-seven patients (48.1 %) filled out their reason (symptom) to visit the ED. Using the Reason for Visit Classification, 201 different reasons were registered. The most common reasons for visiting the ED were injuries and musculoskeletal symptoms, followed by malaise symptoms and abdominal complaints (Table 3).

**Table 3:** Ten most common reasons for visit, classified into categories using The Reason for Visit Classification

Code	Reason for visit category	Number	Percentage
J505-J575	Injury, type unspecified for example ‘foot bumped’, ‘hurt my hand’	356	23.2
J205-J230	Injury, lacerations, and cuts for example ‘cut in finger’	253	16.5
S900-S999	Symptoms referable to the musculoskeletal system, excluding injuries for example ‘low back pain’, ‘stiffness knee’	195	12.7
J800-J899	Injury, not otherwise specified for example ‘motor vehicle accident’, ‘fell from stair cases’	132	8.6
S001-S099	General symptoms for example ‘malaise’, ‘fainting’	113	7.4
S500-S639	Symptoms referable to the digestive system for example ‘abdominal pain’, ‘nausea’	98	6.4
J001-J050	Injury, fractures, and dislocations for example ‘fracture wrist’, ‘dislocated shoulder’	89	5.8
J105-J130	Injury, sprains, and strains for example ‘sprained ankle’, ‘twisted knee’	49	3.2
S400-S499	Symptoms referable to the respiratory system for example ‘shortness of breath’, ‘pain throat’	40	2.6
S300-S399	Symptoms referable to the eyes and ears for example ‘red eye’, ‘pain ear’	33	2.1

Fifteen hundred sixty one patients (48.8 %) answered the question why they attended the ED without a referral. Table 4 shows the distribution of the given answers. The three most chosen reasons were the following: the expectation to get help faster going directly to the ED (437 patients, 28.0 %), the expectation to need radiologic or laboratory investigations (372 patients, 23.8 %), and the presumption that the symptoms were too severe to visit a GP (355 patients, 22.7 %). When focusing on appropriateness in relation to these answers, it is notable that patients expecting investigations to be necessary or thinking their symptoms are too severe to visit a GP significantly more often appropriately visited the ED. Patients that were from a different region were significantly less often classified as appropriately visiting the ED.

**Table 4:** Answers to the question why self-referred patients directly attended the ED

Multiple choice answer	Number (%)	Appropriate (%)	Inappropriate (%)	*P* value
Patients that answered this question	*1561*	*908 (58.2)*	*653 (41.8)*	
Takes less time	437 (28.0)	250 (27.5)	187 (28.6)	0.632
Investigations necessary	372 (23.8)	246 (27.1)	126 (19.3)	*<0.001*
Symptoms too severe	355 (22.7)	227 (25.0)	128 (19.6)	*0.012*
Not from the region	258 (16.5)	131 (14.4)	127 (19.4)	*0.008*
GP not available	145 (9.3)	83 (9.1)	62 (9.5)	0.812
GP could/would not see me	130 (8.3)	65 (7.2)	65 (10.0)	0.049*
No confidence in GP	47 (3.0)	30 (3.3)	17 (2.6)	0.424
No GP	20 (1.3)	8 (0.9)	12 (1.8)	0.097

Patients could choose more than one answer

*Number (%)* number of patients that chose this answer (percentage), *Appropriate (%)* number of patients that chose this answer, whose visit was considered appropriate (percentage of patients with an appropriate visit that chose this answer), *Inappropriate (%)* number of patients that chose this answer, whose visit was considered inappropriate (percentage of patients with an inappropriate visit that chose this answer)

The data in italics represent signifant P-values *After applying the Benjamini-Hochberg method, this *P* value is no longer significant

Patients could also choose to give an open answer to this question; this was done by 191 patients. Different answers, that were not a multiple choice option, were given: ‘The concierge send me to the hospital’, ‘I want more specific help, like stitches, injections etc., ‘My GP will send me to the ED anyway’, and ‘I did not want to take any risk’.

Fourteen hundred six patients (44.0 %) answered the question where they would go the next time they are suffering from similar symptoms. The majority, 1104 patients (78.5 %), answered they would again turn primarily to the ED, 320 patients (22.8 %) would visit a GP or a GP cooperative next time, and 16 patients (1.1 %) would seek no medical help at all. Some patients selected multiple answers to this question. When appropriateness was taken into account in relation to these answers, it was found that patients returning to the ED were significantly more often appropriately visiting the ED, whereas patients that would turn to their GP or seek no medical help were significantly more often inappropriately visiting the ED (Table 5).

**Table 5:** Answers to the question where self-referred patients would seek medical help, confronted with similar symptoms

Multiple choice answer	Number (%)	Appropriate (%)	Inappropriate (%)	*P* value
ED	1104 (78.5)	686 (62.1)	418 (37.9)	<0.001
GP	320 (22.8)	144 (45.0)	176 (55.0)	<0.001
No medical help	16 (1.1)	5 (31.3)	11 (68.8)	0.029

The Benjamini-Hochberg method was applied on the tests shown in Tables 2, 4, and 5. After this correction, the *P* value 0.049 is no longer significant (Table 4: GP could/would not see me).

## Discussion

The present study used questionnaires to explore motives of self-referred patients visiting the ED. It is remarkable to see that the reasons for self-referral are similar even in the Netherlands, which has a well-developed primary care system.

This study found that the main reason for most patients to skip a visit to their GP and go straight to the ED is the expectation that they would get medical help sooner. Several patients answered that it is devious to visit a GP or GP cooperative first, to be referred to an ED ‘anyway’. This is mostly true in a situation where the GP cooperative and ED are not closely situated, like it is the case in the hospital this study was conducted in. Previous research also found that time is playing a major role in choosing to attend an ED [3, 9, 15]. Many self-referrals responded that their symptoms were too severe to visit a GP. This is consistent with earlier studies showing that health concerns and the belief of having an urgent medical problem play a major role in deciding to attend an ED [7–13, 16]. Furthermore, this study, in concordance with previous research, found that patients often are convinced that they need radiologic or laboratory investigations to get a diagnosis [3, 6, 16]. It therefore seems a logical step to attend to the ED directly, where it is possible to get these investigations. Consistent with previous literature, this study found that patients are frequently supported in their decision to visit an ED by family members or paramedics [6, 11]. The majority of the self-referred patients visited with injuries and other symptoms of the musculoskeletal system. Our results are again consistent with previous studies, showing that injuries and musculoskeletal symptoms are frequent reasons to attend an ED [3, 6, 11, 17].

Multiple non-Dutch studies found that the unavailability of a GP is a major reason to self-refer to an ED, especially after hours [7, 8, 15, 16]. The present study found that this was a reason to attend the ED for almost a fifth of the self-referrals. This result, however, is in contrast with previous Dutch studies on this subject, stating that problems in consulting a GP were not often a reason to self-refer [3, 18]. This discrepancy is interesting. In the Netherlands, the primary healthcare system is well organized: patients can visit their own GP in daytime and, with the continuing development of GP cooperatives since the mid-1990s, they have a perceived easy access to primary care in the evening and night as well. The present study shows that Dutch patients nonetheless are having difficulties in gaining an appointment with a GP in a timely manner. This might be caused by increasingly busy general practices and enlarging GP cooperatives taking care of growing numbers of patients, leading to more bureaucracy and stricter regulations for getting an appointment. In addition, the modern patient seems to expect and demand medical care at the moment he/she thinks this is mandatory, and is increasingly less willing to wait for an appointment.

When concentrating on the appropriateness in relation to the answers patients selected, it seems that patients do have a sense of when to visit the ED for their symptoms. Patients visiting the ED because of health concerns are more often visiting the ED appropriately than patients visiting out of practical reasons. To the best of our knowledge, there are no previous studies looking at the motives of self-referred patients for visiting the ED in relation to the appropriateness of their visits.

### Limitations

This study made use of a questionnaire that was not validated. However, to the best of our knowledge, there is no validated questionnaire regarding this subject. The response rate to the questionnaire was 48.9 %, which is relatively low. This makes it possible that the included answers are not a reflection of the opinion of all self-referred patients.

This study made use of predefined criteria to determine whether an ED visit was appropriate. This method can lead to an overestimation of the number of appropriate visits because it is possible that physicians working in the ED order more investigations than a GP would with the same patient. In our previous study, we also used diagnoses and treatments given to the included patients to determine appropriateness [4]. With this method, 48.1 % of the self-referrals was found appropriate versus 58.8 % using the predefined criteria. In order to make the current study not too complicated, the choice was made to include only the predefined criteria.

Another limitation of this study is the possibility of interobserver bias. Different physicians working in the ED may order different investigations with similar symptoms, which can lead to different outcomes using the predefined criteria. These individual variations are not completely avoidable, and the effect on the percentage of appropriateness is not clear. This study was performed in a single ED. This limits the possibility to extrapolate the results to other EDs in the Netherlands or other countries.

## Conclusions

This study, carried out in a community hospital in the Netherlands, found that the choice of patients to self-refer to an ED is often a considered decision. Patients are looking for specialist help for their perceived urgent symptoms and want fast and easy access to radiologic and laboratory investigations. While the Netherlands has a well-developed primary care network, the reasons for self-referral in the Netherlands are similar to reasons found in previous literature based in other countries. Despite the strong primary care, Dutch patients report difficulties in gaining a timely appointment with a GP. Patients visiting the ED out of health concerns are more often visiting appropriately versus patients visiting for more practical reasons.
